# The Presence of Tertiary Lymphoid Structures Provides New Insight Into the Clinicopathological Features and Prognosis of Patients With Breast Cancer

**DOI:** 10.3389/fimmu.2022.868155

**Published:** 2022-05-19

**Authors:** Bin Wang, Jie Liu, Yin Han, Yaotiao Deng, Jinze Li, Yu Jiang

**Affiliations:** ^1^ Department of Medical Oncology, Cancer Center, West China Hospital, Sichuan University, Chengdu, China; ^2^ Department of Pathology, Chengdu Fifth People’s Hospital, Chengdu, China; ^3^ Department of Urology, Institute of Urology, West China Hospital, Sichuan University, Chengdu, China

**Keywords:** tertiary lymphoid structures, breast cancer, prognosis, survival, clinicopathological parameters, signature

## Abstract

**Background:**

Tertiary lymphoid structures (TLSs) have been proven to be predictive biomarkers of favorable clinical outcomes and response to immunotherapies in several solid malignancies. Nevertheless, the effect of TLSs in patients with breast cancer (BC) remains controversial. The objective of the current study is to investigate the clinicopathological and prognostic significance of TLSs in BC. Given the unique difficulties for detecting and quantifying TLSs, a TLS-associated gene signature based on The Cancer Genome Atlas (TCGA) BC cohort was used to validate and supplement our results.

**Methods:**

Electronic platforms (PubMed, Web of Science, EMBASE, the Cochrane Library, CNKI, and Wanfang) were searched systematically to identify relevant studies as of January 11, 2022. We calculated combined odds ratios (ORs) with 95% confidence intervals (CIs) to determine the relationship between clinicopathological parameters and TLSs. The pooled hazard ratios (HRs) and 95% CIs were also calculated to evaluate the prognostic significance of TLSs. The TLS signature based on the TCGA BC cohort was applied to validate and supplement our results.

**Results:**

Fifteen studies with 3,898 patients were eligible for enrollment in our study. The combined analysis indicated that the presence of TLSs was related to improved disease-free survival (DFS) (HR = 0.61, 95% CI: 0.41–0.90, *p* < 0.05) and overall survival (OS) (HR = 1.66, 95% CI: 1.26–2.20, *p* < 0.001). Additionally, the presence of TLSs was positively correlated with early tumor TNM stage and high tumor-infiltrating lymphocytes. TLS presence was positively related to human epidermal growth factor receptor 2 (HER-2) and Ki-67 but inversely correlated with the status of estrogen and progesterone receptor. Simultaneously, our study found that tumor immune microenvironment was more favorable in the high-TLS signature group than in the low-TLS signature group. Consistently, BC patients in the high-TLS signature group exhibited better survival outcomes compared to those in the low-TLS signature group, suggesting that TLSs might be favorable prognostic biomarkers.

**Conclusions:**

TLS presence provides new insight into the clinicopathological features and prognosis of patients with BC, whereas the factors discussed limited the evidence quality of this study. We look forward to consistent methods to define and characterize TLSs, and more high-quality prospective clinical trials designed to validate the value of TLSs alone or in combination with other markers.

## Introduction

Breast cancer (BC) has been the most frequently diagnosed malignancy worldwide, and is the main cause of tumor-associated mortality in women (1, 2). Originating from mammary epithelial cells, BC as a kind of heterogeneous disease has divergent histological subtypes and biological characteristics, thus leading to distinct clinical behaviors and treatment sensitivity profiles (3). Although the recent success of immunotherapy has paved the way for various solid or hematological malignancies, most subtypes of BC exhibit little efficacy to immunotherapy with immune checkpoint inhibitors only approved in combination therapy for PD-L1-positive metastatic triple-negative breast cancer (TNBC) (4). Poor immunogenicity, lack of T-cell infiltration, and an immunosuppressive tumor microenvironment (TME) have been identified as major barriers to the success of immunotherapy in BC (4). The interaction between tumor cells and the immune TME is a complex, dynamic, and evolving process; thus, conventional tumor characteristics and biomarkers may not be adequate to predict immunotherapy effectiveness and prognostication. Data across large BC clinical trials supported that the high levels of tumor-infiltrating lymphocytes (TILs) are predictive biomarkers for favorable prognosis and of the response to immunotherapy, particularly in HER-2^+^ BC and TNBC (5). Besides TILs, recent evidence revealed that spatial organization plays a crucial role in determining prognosis and response to immunotherapy, with tertiary lymphoid structures (TLSs) attracting widespread attention (6, 7).

TLSs are ectopic cellular aggregates in nonlymphoid tissues under conditions of chronic inflammation including tumors, and share similar architectural and functional characteristics with secondary lymphoid organs (SLOs) (8). The architecture of mature TLSs is characterized by B-cell-enriched zones that consists of B-cell follicles surrounded by a network of follicular helper T cells and follicular dendritic cells, T-cell-enriched regions with dendritic cells (DCs), high endothelial venules (HEVs), as well as lymphatic vessels (6, 7). In addition to the relevant number of immune cells, TLSs emphasize the spatial proximity of specialized subsets of immune cells within TLSs. In contrast to SLOs, TLSs represent privileged sites for local lymphocyte differentiation and antigen presentation, which provide an important milieu for both cellular and humoral antitumor immunity (7). Accumulating research has indicated that TLS presence was deeply associated with positive immunoreactivity and favorable clinical outcomes in most types of solid tumors (6). However, some studies evaluated the prognostic value of TLSs limited to small study numbers and subsets of BC, with inconsistent and conflicting results. Although a previous meta-analysis by Zhang et al. suggested that TLSs were related to better prognosis, their result was based on a limited number of studies, with only two or three studies providing survival outcomes (9). Furthermore, all included studies in their meta-analysis showed that TLSs were beneficial for prognosis, but opposite conclusions have been reported in the recent study (10).

Hence, with the publication of new studies regarding this topic, further evaluation of the role of TLSs in BC is necessary. This study including more than 15 articles aimed to comprehensively assess clinicopathological and prognostic values of TLSs in BC, providing higher-level medical evidence for clinical practice. Simultaneously, given the unique difficulties in the detection and quantification of TLSs, the TLS-related gene signature based on the TCGA BC cohort was further used to validate and supplement our results.

## Materials and Methods

The present study was performed in accordance with the Preferred Reporting Items for Systematic Review and Meta-Analysis (PRISMA) criteria (11). The protocol of this meta-analysis was registered in the PROSPERO (registration number: CRD42022302921).

### Search Strategies

Six electronic platforms (PubMed, Web of Science, EMBASE, the Cochrane Library, CNKI, and Wanfang) were searched systematically to identify eligible studies as of January 2022, regardless of any restrictions in the region or language. Random combinations of the following items were applied in our search: “Tertiary Lymphoid Structure OR tertiary lymphoid organ OR Ectopic Lymphoid Tissue OR Ectopic Lymphoid-Like Structure”, and “breast neoplasm OR breast cancer OR breast tumor OR breast carcinoma”. Additionally, references cited in relevant studies and reviews were manually searched to identify potential studies for inclusion. Two researchers independently reviewed the literature, and any differences were addressed *via* discussion with a third researcher.

### Inclusion and Exclusion Criteria

The eligible studies were selected in accordance with the following criteria: (1) the patients were definitively diagnosed with BC by histopathological examination; (2) TLSs were determined by the hematoxylin and eosin (H&E) staining method or immunohistochemistry (IHC) method based on BC tissues; and (3) studies reported the association of TLS presence with clinicopathological parameters or survival outcomes, including disease-free survival/overall survival (DFS/OS). Exclusion criteria included the following: (1) reviews, editorials, letters, conference abstracts, case reports, or unpublished articles; (2) studies involving animal models or cell lines; (3) studies with unavailable data or insufficient data for analyses; and (4) studies composed of an overlapping patient population.

### Data Extraction

All required data were extracted from eligible studies by two investigators independently, which were as follows: (1) first author, publication date, country, sample size, detection methods, TLS location, cutoff criteria, and study design; (2) clinicopathological parameters, including the association between TLSs and age, tumor size, lymph node status, lymphovascular invasion (LVI), histological grade, TNM stage, estrogen receptor (ER) status, progesterone receptor (PR) status, human epidermal growth factor receptor 2 (HER-2) status, and the cell proliferation marker Ki-67 index; and (3) hazard ratios (HRs) and 95% confidence intervals (CIs) of DFS and OS. If survival outcomes were not given explicitly, the HR with 95% CI was retrieved from Kaplan–Meier curves through Engauge Digitizer (version 4.1) software and Tierney’s reported method (12).

### Quality Evaluation

The quality of the selected studies was independently evaluated by two researchers using the Quality in Prognosis Studies (QUIPS) tool of the Cochrane Prognosis Methods Group, which considers the following domains: (1) study participation, (2) study attrition, (3) prognostic factor measurement, (4) outcome measurement, (5) study confounding, and (6) statistical analysis and reporting (13). Each domain was scored low, moderate, or high risk of bias by answering three to six more detailed questions (**Supplementary Table 1**) (14). Studies were considered of high quality when risk of bias was rated low in at least four of the six domains, and low in both study attrition and study confounding. Any disagreements were resolved by consultation with a third researcher.

### Bioinformatics Analysis

The mRNA expression and clinical information of BC patients in this study were downloaded from the TCGA database (https://portal.gdc.cancer.gov/). We applied single-sample Gene Set Enrichment Analysis (ssGSEA) to quantify the enrichment scores of TLS signature-related genes (CCR6, CD1D, CD79B, CETP, EIF1AY, LAT, PTGDS, RBP5, and SKAP1) (15). We separated patients into three groups equally according to the tertile of the TLS score. The ESTIMATE algorithm was used to analyze the immune score, stromal score, ESTIMATE score, and tumor purity to test the effect of the high- and low-TLS signature groups. The enrichment levels of the 29 immune-associated gene sets were quantified by the ssGSEA score (16), and the relative fractions of 22 human immune cell infiltration were accurately calculated by the CIBERSORT deconvolution algorithm (17), further testing the difference between the high- and low-TLS signature groups using Mann–Whitney *U* test. Correlation analysis between TLS scores and major immune checkpoint genes was performed using Spearman’s algorithm, and the difference in immune checkpoint genes between these two groups was explored by Mann–Whitney *U* test. The survival differences between two groups were compared using a log-rank test, and visualized by Kaplan–Meier curves.

### Statistical Analysis

All calculations were conducted using STATA version 17.0 and R version 4.1.1 with corresponding packages. The pooled odds ratios (ORs) and the corresponding 95% CIs were calculated to assess the association between TLS presence and clinicopathological parameters. The merged HRs with 95% CIs were adopted to evaluate the correlation between TLS presence and prognosis. Heterogeneity between studies was assessed using Cochran’s *Q* and Higgins *I* ^2^ tests. *I* ^2^ > 50% and *p* < 0.10 were defined as significant heterogeneity, and the random-effect model was applied; otherwise, the fixed-effect model was utilized. We conducted a subgroup analysis to investigate the heterogeneity cause. Moreover, sensitivity analysis was employed to assess the stability of the pooled outcomes by dropping each study individually. Meanwhile, both Begg’s funnel plots and Egger’s tests were adopted to evaluate potential publication bias. Statistical significance was defined as a *p*-value of less than 0.05.

## Results

### Study Characteristics

**Figure 1:**
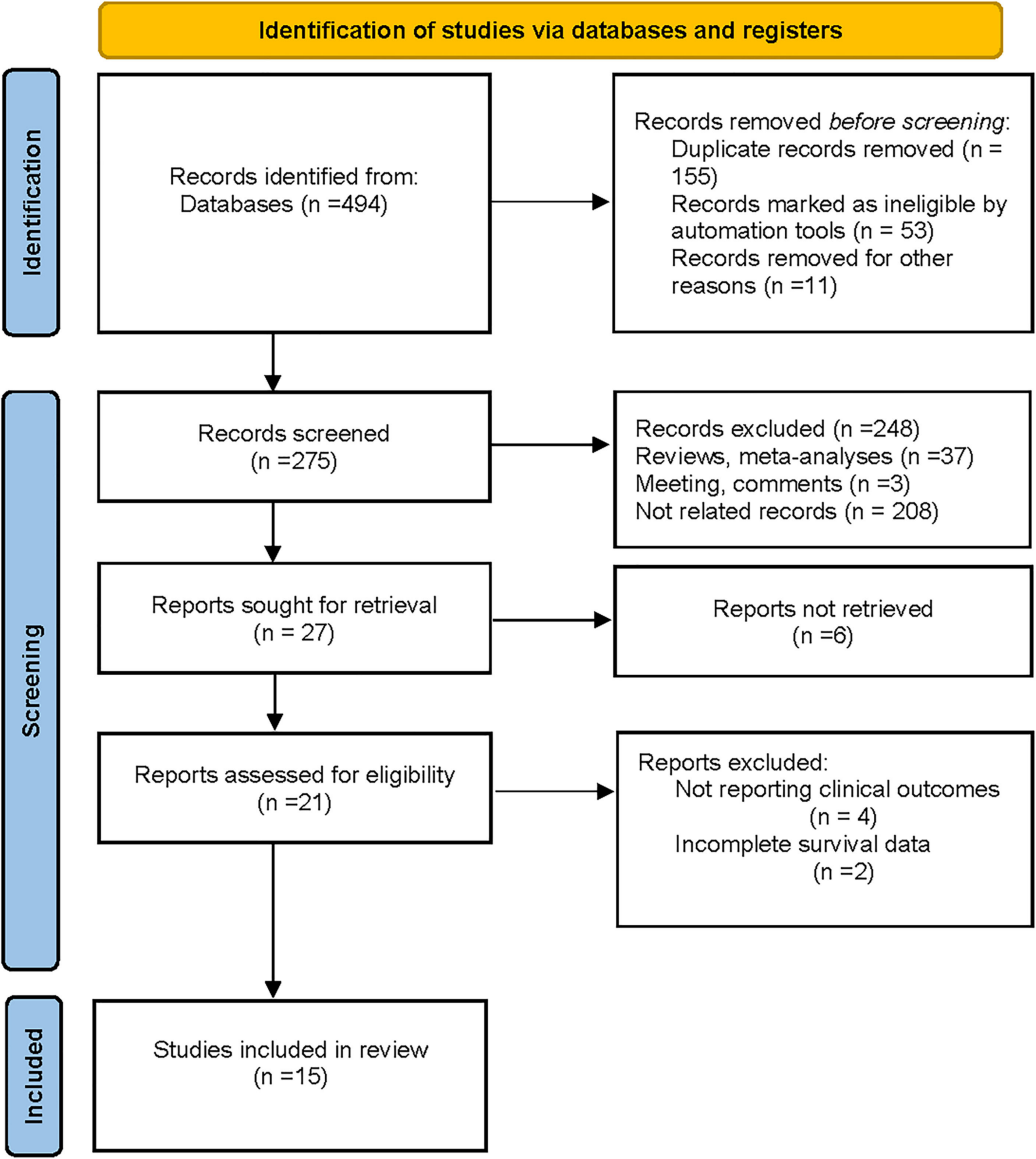
Study search and selection process flow diagram (PRISMA 2020).

**Figure 2:**
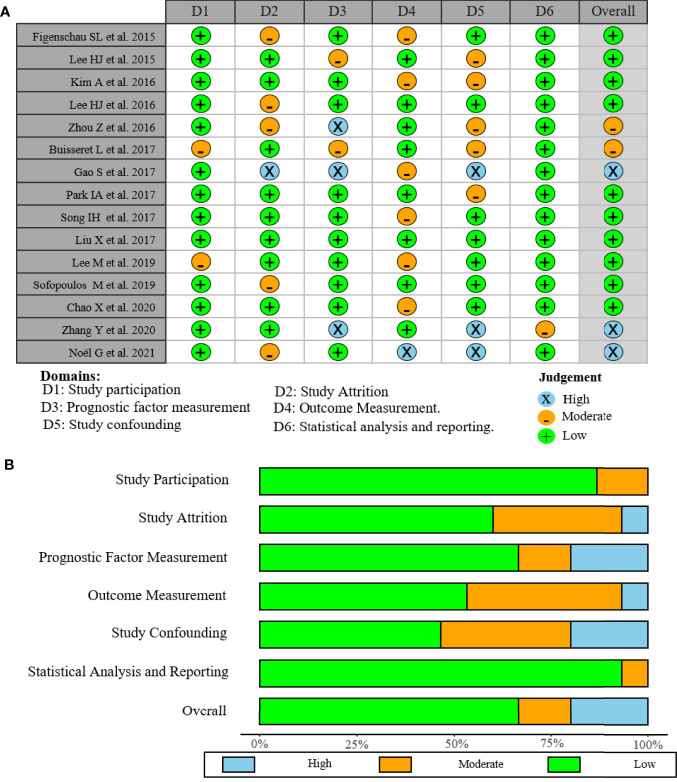
Risk of bias graph of included studies. **(A)** Assessment regarding each risk of bias item for each included study. **(B)** Each bias risk item was presented as a percentage for all included studies.

As shown in the PRISMA flowchart (**Figure 1**), a total of 494 articles were identified from electronic databases according to the initial search strategy. After preliminary screening and full-text review, 15 studies with a total of 3,898 patients (10, 18–31) were fully in conformity with the screening criteria and were included in this study. The baseline characteristics of the eligible studies are summarized in **Table 1**. The fifteen included studies were retrospective studies published between 2015 and 2021, with a patient population ranging from 60 to 769. Seven studies were performed in Korea (18, 20, 23–25, 27, 28), five in China (19, 22, 29–31), two in Greece (10, 26), and one in Belgium (21). Ten of the 15 included studies reported the correlation between clinicopathological features and TLSs (TNM stage, 4 studies; age, 5 studies; tumor size, 4 studies; lymph node status, 7 studies; LVI, 4 studies; histological grade, 7 studies; TILs, 3 studies; ER, 3 studies; PR, 3 studies; HER-2, 6 studies; Ki-67, 2 studies). Ten of the 15 included studies investigated the prognostic role of TLS presence, with eight assessing DFS and four assessing OS. The study quality assessment results of each study using the QUIPS tool suggested that the methodology of the studies was relatively reliable, and only two studies harbored a high overall risk of bias (**Figure 2**).

**Table 1:** Main characteristics of the eligible studies.

Eligible study	Year	Country	Sample size	Median age (range)	Cohort	Detected method	TLS markers	TLS location	Cutoff criteria	Survival outcome	Source of HR	Study design
Lee HJ et al. (25)	2015	Korea	447	NR	HER2+ BC	H&E	NA	Within 5 mm from the invasive or *in situ* carcinoma	None, minimal, moderate, or abundant	DFS	Reported	Retrospective
Figenschau SL et al. (26)	2015	Greece	167	NR	PBC	H&E/IHC	CD3, CD4, CD8, CD20, CD21, BCL-6, and PNAd	Global	Very low, low, medium, and high	DFS, OS	Reported	Retrospective
Lee HJ et al. (27)	2016	Korea	769	47 (23–76)	TNBC	H&E/IHC	MECA-79	In tumor adjacent tissue	None, little, moderate, or abundant	DFS, OS	Reported	Retrospective
Kim A et al. (18)	2016	Korea	204	48 (27–76)	Ductal BC	H&E/IHC	CD3 and CD20	Near to or remote from the invasive or *in situ* carcinoma	Absent, low, moderate, or abundant	NR	Reported	Retrospective
Zhou Z et al. (30)	2016	China	100	49.3 (31–72)	PBC	H&E/IHC	CD3, CD20, CD21, BCL-6, and CD62L	Global	Positive vs. negative	NR	Reported	Retrospective
Song IH et al. (23)	2017	Korea	108	42 (23–70)	TNBC	H&E/IHC	CD3, CD8, and CD20	Global	No, little, moderate, or abundant	DFS	Reported	Retrospective
Park IA et al. (20)	2017	Korea	681	47.4 (23–76)	TNBC	H&E	NA	In the adjacent area of the invasive and *in situ* carcinoma	Absent, low, moderate, or abundant	DFS	Reported	Retrospective
Liu X et al. (19)	2017	China	248	NR	Invasive BC	H&E/IHC	CD3, CD20, and CD23	Within 5 mm from the invasive or *in situ* carcinoma	Positive vs. negative	DFS, OS	Survival curve	Retrospective
Buisseret L et al. (28)	2017	Belgium	189	NR	PBC	H&E/IHC	CD3, CD4, CD8, CD20, and CD23	Global	Positive vs. negative	NR	Reported	Retrospective
Gao S et al. (29)	2017	China	150	48.5 (34–75)	Invasive ductal BC	H&E/IHC	CD3, CD4, CD8, CD20, CD21, CD62L, and, BCL-6	Global	Positive vs. negative	NR	Reported	Retrospective
Lee M et al. (24)	2019	Korea	335	NR	Metastatic BC	H&E	NA	Primary and metastatic sites	Present vs. absent	OS	Reported	Retrospective
Sofopoulos M et al. (10)	2019	Greece	167	53 (26–78)	Invasive ductal BC	H&E/IHC	CD3, CD4, CD8, CD20, CD23, CD31, CD163, and, FOXP3	Within 5 mm from the infiltrative tumor border	Negative, low to moderate, and high	DFS/OS	Survival curve	Retrospective
Chao X et al. (22)	2020	China	60	50 (25–81))	Metaplastic BC	H&E/IHC	CD3 and CD20	Within the invasive border	Absent and present	DFS	Reported	Retrospective
Zhang Y et al. (31)	2020	China	105	52 (30–79)	Invasive ductal BC	H&E/IHC	CD3, CD10, CD20, and CD21	Within 5 mm from the invasive or *in situ* carcinoma	Absent and present	NR	Reported	Retrospective
Noël G et al. (21)	2021	Belgium	168	NR	Invasive ductal BC	H&E/IHC	CD3 and CD20	Global	No, inactive, and active	DFS	Survival curve	Retrospective

BC, breast cancer; TNBC, triple-negative breast cancer; DFS, disease-free survival; OS, overall survival; NR, not reported; NA, not applicable; H&E, hematoxylin and eosin staining; IHC, immunohistochemistry.

### Correlation Between TLS Presence and Clinicopathological Parameters

**Figure 3:**
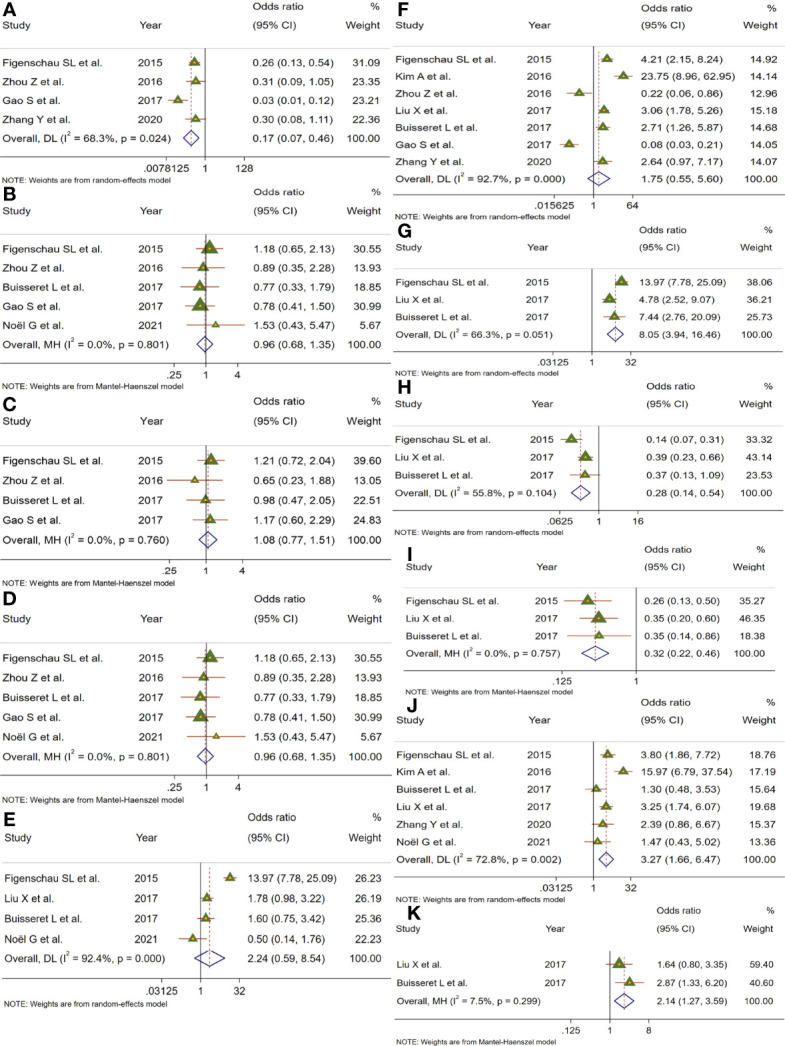
Meta-analysis for the association of TLSs with clinicopathological parameters. Forest plots showed the correlation between TLS presence and **(A)** TNM stage, **(B)** age, **(C)** tumor size, **(D)** lymph node status, **(E)** lymphovascular invasion, **(F)** histological grade, **(G)** TILs, **(H)** ER, **(I)** PR, **(J)** HER-2, and **(K)** Ki-67. Each result was shown by the OR with 95% CI. Diamonds indicated pooled OR with their corresponding 95% CIs.

To evaluate the value of TLSs as an effective biomarker, we investigated the relationship between the TLS presence and certain clinicopathological parameters in patients with BC. The results of this analysis are shown in **Figure 3** and **Supplementary Table 2**. The pooled OR revealed that TLS presence was more prevalent in BC patients with earlier tumor TNM stage (OR = 0.17, 95% CI: 0.07–0.46, *p* < 0.001; *I* ^2^ = 68.3%, *p* = 0.024) (**Figure 3A**). However, the correlation between TLS presence and age (OR = 0.96, 95% CI: 0.68–1.35, *p* = 0.802; *I* ^2^ = 0%, *p* = 0.800), tumor size (OR = 1.08, 95% CI: 0.77–1.51, *p* = 0.680; *I* ^2^ = 0%, *p* = 0.760), lymph node status (OR = 0.64, 95% CI: 0.31–1.30, *p* = 0.215; *I* ^2^ = 86.6%, *p* < 0.001), LVI (OR = 2.25, 95% CI: 0.59–8.54, *p* = 0.236; *I* ^2^ = 92.4%, *p* < 0.001), and histological grade (OR = 1.75, 95% CI: 0.55–5.60, *p* = 0.346; *I* ^2^ = 92.7%, *p* < 0.001) was not statistically significant (**Figures 3B–F**). TLSs have recently drawn attention as markers for TILs. The pooled results from three included studies showed that TLS presence was positively associated with TILs in tumors (OR = 8.054, 95% CI: 3.94–16.46, *p* < 0.001; *I* ^2^ = 66.3%, *p* = 0.051) (**Figure 3G**). Moreover, a total of 8 studies investigated the correlation of TLS presence with the expression of immunohistochemical markers (ER, PR, HER-2, and Ki-67) (**Figures 3H–K**). The pooled results showed that TLS presence was negatively associated with the expression of ER (OR = 0.28, 95% CI: 0.14–0.54, *p* < 0.001; *I* ^2^ = 55.8%, *p* = 0.104) and PR (OR = 0.318, 95% CI: 0.22–0.47, *p* < 0.001; *I* ^2^ = 0%, *p* = 0.757). In addition, TLS was correlated with high expression of HER-2 (OR = 3.27, 95% CI: 1.66–6.47, *p* = 0.001; *I* ^2^ = 72.8%, *p* = 0.002) and Ki-67 (OR =2.14, 95% CI: 1.27–3.59, *p* < 0.004; *I* ^2^ = 7.5%, p = 0.299).

### Effect of TLS on Survival Outcomes of Patients With Breast Cancer

**Figure 4:**
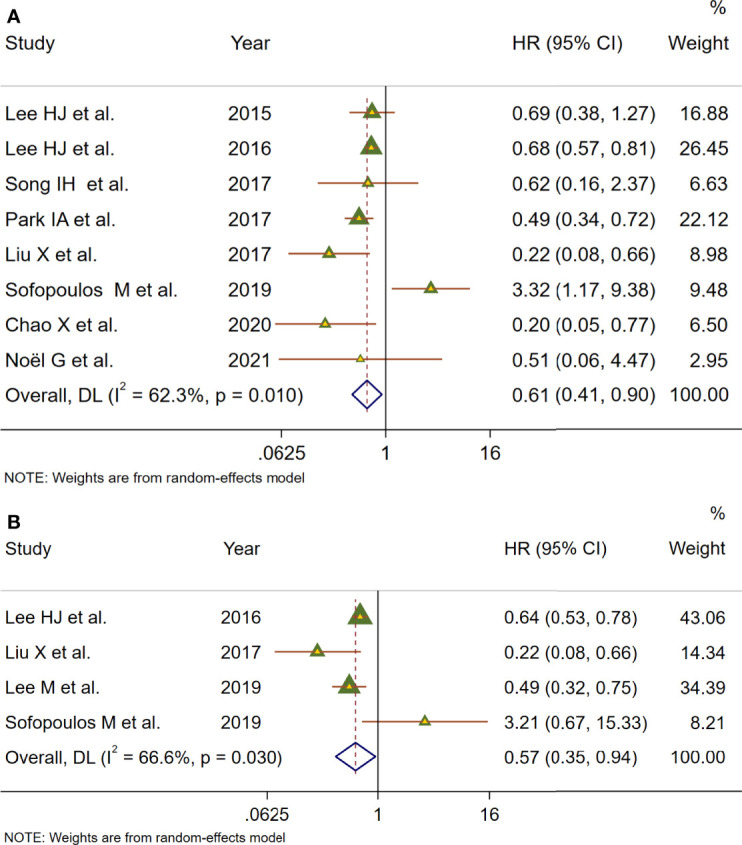
Meta-analysis of the prognostic value of TLS presence in BC patients. **(A)** Forest plots of the association between the TLS presence and disease-free survival. **(B)** Forest plots of the association between the TLS presence and overall survival. An HR <1 suggested that the presence of TLSs was associated with favorable prognosis. Diamonds indicated overall HR with their corresponding 95% CIs.

To deeply assess the prognostic effect of TLSs in BC patients, a meta-analysis was performed on HRs for DFS and OS. Eight studies with 572 patients examined the relationship between TLS presence and DFS (**Figure 4A**). Because of moderate heterogeneity between included studies (*I* ^2^ = 62.3%, *p* = 0.010), a random-effect model was performed to evaluate the pooled HR and 95% CI of DFS. The merged results suggested that TLS presence was obviously related to a better DFS (HR = 0.61, 95% CI: 0.41–0.90, *p* < 0.05). Four studies including 1,666 patients assessed the association between TLS presence and OS (**Figure 4B**). Since heterogeneity across studies was *I* ^2 =^ 52.9%, *p* = 0.038, a random-effect model was adopted for analysis. The merged results indicated that TLS presence was correlated with longer OS (HR = 1.66, 95% CI: 1.26–2.20, *p* < 0.001).

### Subgroup Analyses

Limited to the number of studies included, we only performed subgroup analysis for DFS and stratified by median age, ethnicity, sample size, source of data, and detection method (**Table 2**). The DFS rate did not differ between patients with a median age below 50 years and those over 50 years and between sample sizes greater than or less than 300. Subgroup analysis stratified by ethnicity and source of data showed that TLS expression in both Asian and univariate analyses was more prone to be correlated with better DFS (HR = 0.63, 95% CI: 0.54–0.73, *p* < 0.001) with low heterogeneity (*I* ^2^ = 43.2%, *p* = 0.117). Nevertheless, for two studies in Caucasians, the pooled data reached the opposite conclusion (HR = 1.67, 95% CI: 0.29–9.80, *p* = 0.924) with significant heterogeneity. For subgroup analyses based on the detection method, the results suggested that TLS presence predicted better DFS with detection using H&E staining (HR = 0.61, 95% CI: 0.45–0.82, *p* < 0.001), while TLS detected by H&E staining combined with IHC had no statistically significant correlation for DFS (HR = 0.29, 95% CI: 0.26–1.37, *p* = 0.224). Thus, ethnicity, source of data, and/or detection method might be a source of heterogeneity. Moreover, the heterogeneity among studies might be caused by the complex subtypes of BC.

**Table 2:** Subgroup analysis of the prognostic value of TLSs for DFS in patients with breast cancer.

Subgroup analysis	No. of studies	Effect model	Pooled HR (95%CI)	*p*	Heterogeneity	
					*I* ^2^(%)	*p*
DFS						
Total	8	Random	0.61 (0.41, 0.90)	0.013	62.3	0.010
Median age						
<50	3	Fixed	0.64 (0.55, 0.75)	<0.001	13.8	0.314
≥50	3	Random	0.54 (0.08, 3.57)	0.524	87.6	0.000
Ethnicity						
Asian	6	Fixed	0.63 (0.54, 0.73)	<0.001	43.2	0.117
Caucasian	2	Random	1.67 (0.29, 9.80)	0.568	57.0	0.127
Sample size						
<300	5	Random	0.62 (0.35, 1.10)	0.104	75.2	0.003
≥300	3	Fixed	0.64 (0.53, 0.77)	<0.001	15.8	0.305
Source of data						
Univariate	6	Fixed	0.63 (0.54, 0.73)	<0.001	43.2	0.117
K-M curves	2	Random	1.67 (0.29, 9.80)	0.568	57.0	0.127
Detected method						
H&E	3	Random	0.61 (0.45, 0.82)	0.001	56.9	0.128
H&E and IHC	5	Random	0.29 (0.26, 1.37)	0.224	69.2	0.006

HR, hazard ratio; CI, confidence interval; H&E, hematoxylin and eosin staining; IHC, immunohistochemistry.

### Sensitivity Analysis

**Figure 5:**
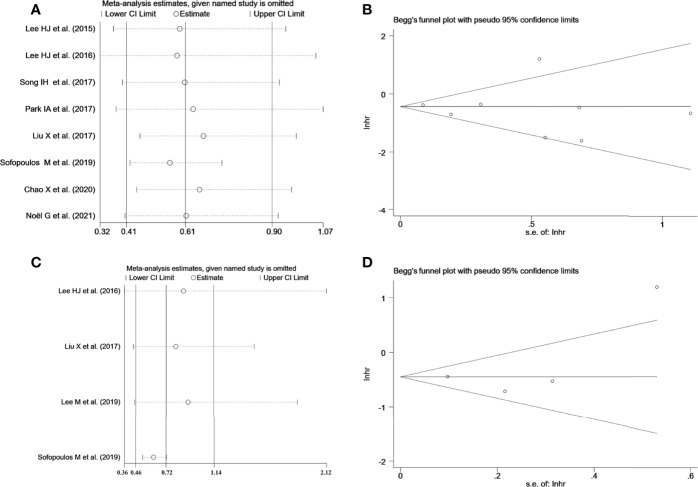
**(A)** Sensitivity analysis between TLS presence and DFS. **(B)** Sensitivity analysis between TLS presence and OS. **(C)** Begg’s funnel plot for publication bias of TLS presence on DFS. **(D)** Begg’s funnel plot for publication bias of TLS presence on OS.

Sensitivity analysis was employed to investigate the stability of the pooled survival outcomes by sequentially dropping each study individually (**Figures 5A, B**). The final result indicated that no significant influence of the merged survival outcomes was observed after removing any of the included studies, demonstrating that our results were stable and reliable.

### Publication Bias

Both Begg’s funnel and Egger’s tests were conducted to estimate the potential publication bias. Begg’s funnel plots appeared symmetrical (Begg’s: *p* = 0.386 for DFS; *p* = 0.734 for OS), and the *p*-values in Egger’s test were 0.701 for DFS and 0.529 for OS, As shown in **Figures 5C, D**. Thus, there was no significant publication bias in studies on TLSs with respect to survival analysis.

### Validation Results of the TLS Signature Based on The Cancer Genome Atlas

**Figure 6:**
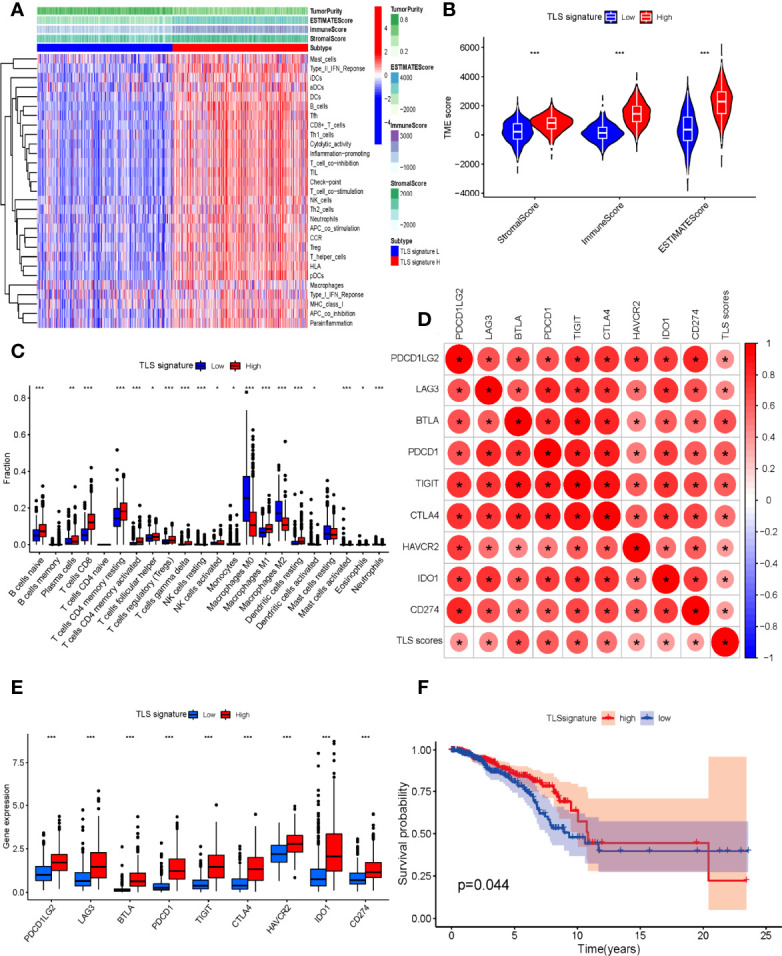
**(A)** Relationship between TLS signature and tumor immune microenvironment. Twenty-nine immune-associated gene sets were quantified by ssGSEA. Tumor purity, estimate scores, stromal scores, and immune scores were evaluated by ESTIMATE. **(B)** Comparison of stromal scores, immune scores, and ESTIMATE scores between the high- and low-TLS signature groups (Mann–Whitney *U* test). **(C)** The relative fractions of 22 human immune cell infiltration in the high- and low-TLS signature groups (Mann–Whitney *U* test). **(D)** The correlation between TLS signature scores and immune-related checkpoint gene expression (Spearman’s test). **(E)** Comparison of immune-related checkpoint genes between the high- and low-TLS signature groups (Mann–Whitney *U* test). **(F)** Comparison of OS between the high- and low-TLS signature groups (log-rank test). *p < 0.05, **p < 0.01, ***p < 0.001.

At present, the major research dilemma for TLSs is lack of standards for detection and quantification. Detecting TLSs through H&E staining and IHC is susceptible to subjective bias and inconvenient for quantifying TLSs. Recently, several gene signatures detecting TLSs identified from transcriptomic analysis were proven to be feasible in the quantification of TLSs. The 9-gene TLS signature mainly represented the B cells and T cells in TLSs, which was thought to be more representative of TLS-associated gene expression than the 12-chemokine signature (32). The 9-gene signature has been used for TLS quantification in a variety of solid tumors such as lung adenocarcinoma and melanoma, conveying significant prognostic and predictive value (15, 32). First, we comparatively assessed the differential expression of 9 genes between tumor and normal tissues in the TCGA BC cohort (**Figure S1**). Based on the 9-gene enrichment score, BC patients were separated into a high-TLS signature group (top tertile) and a low-TLS signature group (bottom tertile). We then investigated correlations between the expression of the 9-gene signature and the TME. In the ESTIMATE algorithm, patients in the high-TLS signature group had higher immune, stromal, and ESTIMATE scores and lower tumor purity than patients in the low-TLS signature group (**Figures 6A, B**). As shown in **Figure 6A**, the infiltration degree of immune cell subsets in the high-TLS signature group was significantly higher than that in the low-TLS signature group. The CIBERSORT analysis indicated that the relative proportions of immune cells including B cells, plasma cells, CD8 T cells, CD4 T cells, follicular helper T cells, regulatory T cells (Tregs), NK cells, monocytes, macrophages, activated dendritic cells, mast cells, neutrophils, and eosinophils were significantly different between the high- and low-TLS signature groups (**Figure 6C**). The differences in immune cell proportion indicated that the 9-gene signature can efficiently reflect the enrichment of TLSs in the TME. We next evaluated the correlation between the TLS signature and the expression of immune-related checkpoint genes. Pearson correlation analysis revealed that the TLS signature score was positively correlated with immune-related checkpoint expression. Notably, compared with the low-TLS signature group, the expression of all major checkpoint genes was significantly upregulated in the high-TLS signature group. We then further assessed the prognostic value of the TLS signature in patients with BC. The Kaplan–Meier curve revealed the high-TLS signature group was significantly associated with improved OS.

## Discussion

As a complex network composed of a variety of immune subsets, the tumor immune microenvironment exerts a great impact on immunotherapeutic efficacy and prognosis (33). TLSs have attracted increasing attention as a unique structure of the TME. TLSs not only are prognostic biomarkers of improved clinical outcome among cancer patients but also shape a local and favorable site for generating antitumor humoral and cellular immune responses (6, 8). However, several studies exploring the impact of TLS on prognosis and tumor progression were limited to small study numbers and subsets of BCs, of which the results are conflicting and lack more comprehensive evaluations. To the best of our knowledge, this is the most comprehensive meta-analysis including 15 articles to assess the clinicopathological and prognostic value of TLSs in BC.

The prognosis of BC is well recognized to be influenced by host- and tumor-associated factors (age, tumor size, histological grade, lymph node, hormone and growth receptor status, etc.) (19). First, we synthesized eleven pieces of research to evaluate the correlation between TLSs and clinicopathological parameters in BC (**Figure 3**). Our results suggested that the presence of TLSs was correlated with early TNM stage. Consistent with this, the density of TLSs was also found to be obviously increased in early TNM stage in oral squamous cell carcinoma and NSCLC (34, 35). A positive association was found between the presence of TLSs and TIL levels in our study, which might be associated with TLS function. Being nonencapsulated and close to tumor tissues compared to draining lymph nodes, TLSs facilitate rapid migration of APCs to TLSs and presentation of antigen peptides to T-cell APCs at the site of the tumor (36). Indeed, some studies also reported that TILs were the strongest independent factor predicting TLSs, but not all cases with high TILs showed TLS formation (37). We also found that TLSs were negatively related to ER and PR status, but were correlated with high expression of HER-2 and Ki-67. These results were in line with previous studies, which revealed that increased TILs are inversely related to the expression of ER or PR, and are positive with HER-2 status, the pathologic complete response rate, and improved survival outcomes (25, 38). In the current study, we did not find the relationship between TLSs and age, tumor size, LVI, or histologic grade.

We then systematically evaluated the prognostic impact of TLS presence on BC patients (**Figure 4**). Our meta-analysis describes that HR = 0.68 for OS and HR = 0.54 for DFS, both of which were statistically significant. The study revealed that patients with TLS presence had better survival outcomes regarding DFS and OS. It was worth noting that that sensitivity analyses revealed that our results were reliable and robust, but moderate heterogeneity between included studies was observed in survival outcomes, which can be caused by different baseline characteristics of individual studies. Therefore, subgroup analyses were performed using median age, ethnicity, sample size, source of data, and detection method to explore the potential heterogeneity (**Table 2**). The results revealed that ethnicity, source of data, and/or detection method may be a source of heterogeneity. Therefore, it is worth noting that TLSs are hardly accurately identified by H&E staining alone, and IHC with TLS markers is typically necessary to evaluate TLS characteristics. Moreover, the heterogeneity among studies might be due to the complex subtyping of BC. Recent studies have suggested that maturation degrees and distribution of TLSs are critical to determine the impact of TLSs on prognosis. However, due to a lack of data, subgroup analysis could not be conducted to assess the impact of different maturation degrees and distributions of TLSs on survival outcomes. A high proportion of mature TLSs containing GCs was associated with better prognostic outcome than total TLSs, and the prognostic value of TLSs was lost while GC formation was impaired (39, 40). TLSs could localize to the core of tumor tissues called intratumor TLSs and/or the invasive margin of tumor tissues, known as peritumor TLSs (41). Several studies have indicated that the density of peritumor TLSs is associated with improved prognoses, whereas there are a few opposite results. Sofopoulos et al. described that patients with invasive ductal carcinoma having peritumoral TLSs exhibited worse DFS and OS than patients lacking TLSs (10). High levels of tumor-infiltrating Treg cells observed at the peritumoral areas were demonstrated to be correlated with relapse and death in BC patients (42).

Moreover, given the unique difficulties in TLS detection and quantification, we validated and supplemented the results of our analysis by TLS-related gene signature in BC patients (**Figure 6**). Accumulating evidence has confirmed that TLSs are highly associated with immune cell infiltration, which closely have an impact on the development, progression, and prognosis as well as the treatment of BC (43). Hence, the immune score, stromal score, and ESTIMATE score of BC samples were estimated *via* the ESTIMATE algorithm. Higher immune, stromal, or ESTIMATE scores and lower tumor purity were found in patients of the high-TLS signature group than those in the low-TLS signature group. Simultaneously, we observed that most of the 29 immune subsets, which represented immune cell types, functions, and pathways, in the high group were more abundant compared to the low group. Interestingly, the immunosuppressive subsets like Treg cells, which might lead to poor outcomes, were also higher in the high group. Indeed, immunosuppressive cells are also components of TLSs, and associations of TLSs with immunosuppressive cells have been reported in various solid tumors including BC, lung cancer, and melanoma (15, 42, 44). There was evidence that TLSs in combination with “immunoscore” defined by intratumoral immune cells might provide a comprehensive and most powerful prognosticator. Li et al. found that TLSs combined with CD8^+^ T cells and CD57^+^ NK cells provided a higher predictive prognostic accuracy (45). It was still noteworthy that all major checkpoint genes were obviously upregulated in the high-TLS signature group compared with the low-TLS signature group, suggesting that patients with high expression of TLS signature were more likely to benefit from immunotherapy. A study by Cabrita et al. observed that TLS-rich tumors in particular were related to significantly increased survival after CTLA4 inhibitor on the basis of the TLS signature (15). TLS-rich tumors were more infiltrated by CD8^+^ T cells, and these T cells might be depleted, explaining the correlation between immune checkpoint expression and TLSs and why checkpoint inhibitor might result in productive anti-tumor immunity in TLS-rich tumors (46). Intriguingly, checkpoint inhibitor therapy might also promote the formation of TLSs. Analysis of on-treatment tumor biopsies of urothelial carcinoma and melanoma has shown that tumors of responding patients showed a higher number of TLS-associated B cells relative to matched pretherapy samples after neoadjuvant immune checkpoint blockade (8). All these results demonstrated the significant correlations with TLS signature representing the major component of TLSs, which revealed that the 9-gene signature can efficiently reflect TLS enrichment in the TME. Our study also demonstrated that BC patients with a high TLS signature expression displayed improved survival, which showed that TLS signature could act as a favorable prognostic factor for BC patients. Based on the above results and discussion, multiple measures including chemotherapy, immunostimulants, vaccination, and TLS-associated cytokines and chemokines have been applied to explore the induction of TLS formation (40, 47). Considering some immunosuppressive factors such as regulatory T and B cells that impaired the antitumor of TLSs reported from recent studies, therapeutic strategies to induce TLS formation and maturation while inhibiting immunosuppressive factors might create bright prospects for enhancing tumor immunotherapeutic response (48).

This present study as the most comprehensive meta-analysis provides more substantial evidence for clinicopathological and prognostic significance of TLSs in BC. However, important considerations should be emphasized while interpreting the conclusions of this study. The cellular components, locations, and maturation degrees of TLSs might dictate treatment efficacy, tumor recurrence, and patient survival. The heterogeneity of the means used to quantify TLSs further confound their use in the clinic. Because the number of retrieved studies was not sufficient to be analyzed depending on the detection methods, no restriction was placed on the detection methods. Different scoring methods, scoring systems, and thresholds might lead to different results. Other limitations of our study were also noteworthy. First, partial survival data unavailable in the original article were extracted from Kaplan–Meier curves, which are less reliable than data directly acquired from research. Secondly, compared to multivariate analysis, data from univariate analysis may overestimate the effect sizes. Third, all the research data were derived from Asian and Caucasian patients. Accordingly, the global representation of data is insufficient and lacking. Finally, all studies included were retrospectively conducted and might have inherent structural biases. Therefore, prospective randomized trials are required to validate our results in the future.

In conclusion, TLS presence provides new insight into the clinicopathological features and prognosis of patients with BC. The presence of TLSs might have the potential to predict prognosis of BC patients, whereas factors discussed above limited the evidence quality of this study. We look forward to consistent methods to define and characterize TLSs, and more high-quality prospective clinical trials designed to validate the prognostic and predictive value of TLSs alone or in combination with other markers.

## Data Availability Statement

The original contributions presented in the study are included in the article/**Supplementary Material**. Further inquiries can be directed to the corresponding author.

## Author Contributions

Conception and design: BW, JL, and YJ. Wrote the manuscript: BW and JL. Acquired data: YH, YD, and J-ZL. Analyzed the data: BW. Discussed the results and implications of findings: JL, YD, and YH. Interpretation of data: BW and J-ZL. YJ performed critical evaluation, verification of the manuscript, and supported funding. All authors contributed to the article and approved the submitted version.

## Funding

This work was funded by the National Natural Science Foundation of China (Grant Number 81903181) and the 1·3·5 Project for Disciplines of excellence–Clinical Research Incubation Project, West China Hospital (Grant Number 2021HXFH019).

## Conflict of Interest

The authors declare that the research was conducted in the absence of any commercial or financial relationships that could be construed as a potential conflict of interest.

## Publisher’s Note

All claims expressed in this article are solely those of the authors and do not necessarily represent those of their affiliated organizations, or those of the publisher, the editors and the reviewers. Any product that may be evaluated in this article, or claim that may be made by its manufacturer, is not guaranteed or endorsed by the publisher.
