# Supplementary material for: The Presence of Tertiary Lymphoid Structures Provides New Insight Into the Clinicopathological Features and Prognosis of Patients With Breast Cancer
Source: Front Immunol. 2022 May 19;13:868155. doi: 10.3389/fimmu.2022.868155 (PMC9161084; doi:10.3389/fimmu.2022.868155)
Supplement: Supplementary file 1 [file DataSheet_1.pdf]

Supplementary Table 1. the Quality In Prognosis Studies (QUIPS) tool

Each line is answered as Yes/No/Not available and then scored. Scoring: the lack of "No" was low bias, 1 "No" was moderate and more than 1 "No" was a high risk of bias.

1: participation: The study sample adequately represents the population of interest

Adequate participation in the study by eligible persons
Description of the source of the population of interest
Description of the baseline study sample
Adequate description of the sampling frame and recruitment
Adequate description of the period and place of recruitment
Adequate description of inclusion and exclusion criteria

2: Attrition: The study data is available (not lost to follow up) for the study sample

Adequate response rate for study participants
Description of attempts to collect information on participants that drop out
Reasons for loss to follow up are given
Adequate description of participants lost to follow up
No important differences between those that finished study and those that did not

3: PF measurement: Measured in the same way for all participants

Clear definition of the PF is given
Method of PF measurement is adequately valid and reliable
Continuous variables are reported or appropriate cut off points are used
Methods of measuring the PF is the same for all participants
Adequate proportion of the sample has data for the PF
Appropriate methods of imputation are used for missing PF data

4: outcome measurement: The outcome of interest is measured the same for all patients

A clear definition is given of the outcome
Method of outcome measurement is adequately valid and reliable
Method and setting of outcome measurement is the same for all patients

5: confounding: Important potentially confounding factors are accounted for

All the important confounders are measured
Clear definitions of confounders are given
Measurement of confounders is valid and reliable
Measurement of confounding is the same for all patients
Appropriate methods are used for missing confounding factor data
Important potential confounders are accounted for in the analysis
Important potential confounders are accounted for in the study design

6: Statistical analysis: Stats are appropriate and all primary outcomes are reported

Sufficient presentation of data to assess the adequacy of the analytic strategy
Strategy for model building is appropriate and based on a conceptual framework or model
The statistical model is appropriate for the study
There is no selective reporting of results

*PF: Prognostic factor

Overall:

Low risk: risk of bias was rated low on at least four of the six domains and was rated low for both study attrition and study confounding.

Moderate risk: risk of bias was rated low or moderate on at least four of the six domains and was rated moderate for both study attrition and study confounding.

High risk: risk of bias was rated high on at least four of the six domains and/or was related high for study attrition and study confounding

Supplementary Table 2 Association between TLS expression and clinicopathological parameters

Clinicopathological parameters	No.of studies	No. of patients	Effect model	Pooled OR (95%CI)	p	Heterogeneity I^2(%)	p	Relationship with TLS
Age (⩾50 vs. ＜50)	5	678	fixed	0.96 (0.69, 1.35)	0.802	0.0	0.800	No statistical significance
Tumor size (⩾20 vs. ＜20)	4	596	fixed	1.08 (0.77, 1.51)	0.68	0.0	0.760	No statistical significance
Lymph node status (N+ vs N−)	7	1658	Random	0.64 (0.31, 1.30)	0.215	86.6	＜0.001	No statistical significance
LVI (positive vs. negative)	4	699	Random	2.26 (0.59, 8.54)	0.236	92.4	＜0.001	No statistical significance
Histologic grade (3 vs. 1-2)	7	1152	Random	1.75 (0.55, 5.60)	0.346	92.7	＜0.001	No statistical significance
TNM stage (III-IV vs. I-II)	4	578	Random	0.16 (0.07, 0.46)	＜0.001	68.3	0.024	Negative
ER (positive vs. negative)	3	617	Random	0.28 (0.14, 0.54)	＜0.001	55.8	0.104	Negative

PR (positive vs. negative)	3	534	fixed	0.318 (0.22, 0.47)	< 0.001	0.0	0.757	Negative
HER2 (positive vs. negative)	6	974	Random	3.272 (1.66, 6.47)	0.001	72.8	0.002	Positive
Ki67 (positive vs. negative)	2	365	fixed	2.14 (1.27, 3.59)	0.004	7.5	0.299	Positive
TILs (positive vs. negative)	3	626	Random	8.05 (3.94, 16.46)	< 0.001	66.3	0.051	Positive

Abbreviation: LVI lymphovascular invasion, ER estrogen receptor, PR progesterone receptor, Her2 human epidermal growth factor receptor 2, TILs tumor-infiltrating lymphocytes, OR odds ratio

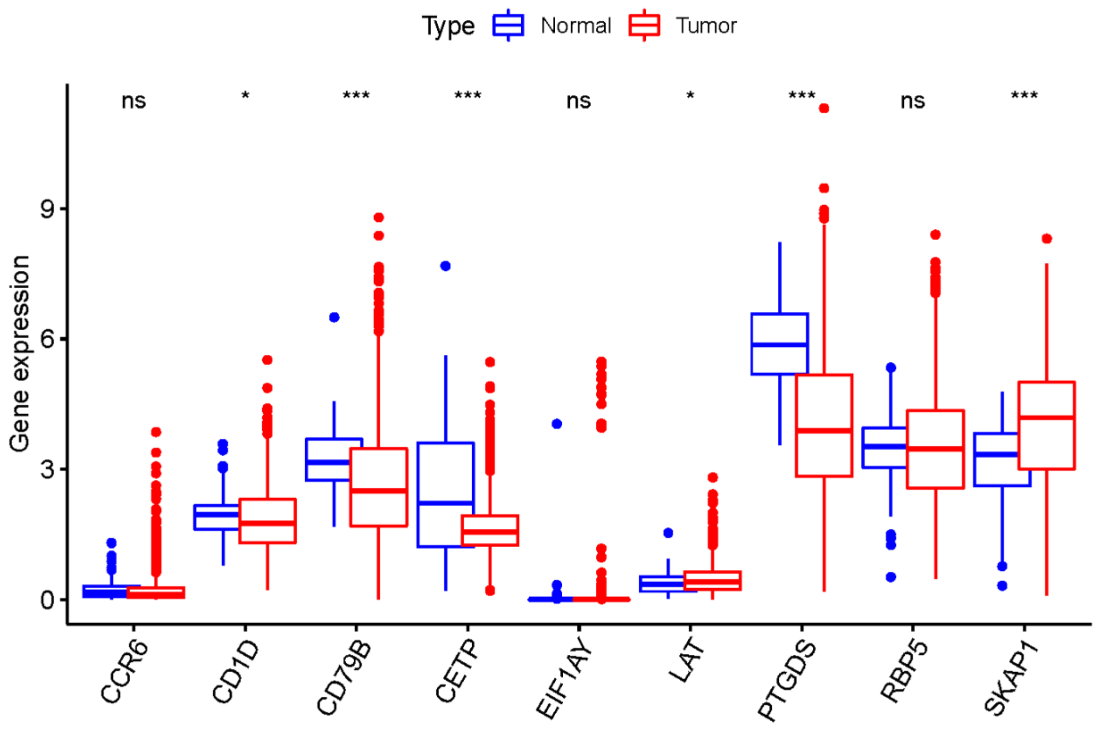

Supplementary Figure 1. the differential expression of 9 genes between tumor and normal tissues
